# Hyperactivity and Risk for Dysregulation of Mood, Energy, and Social Rhythms Syndrome (DYMERS): Standardization of a Simple One-Item Screener versus the Mood Disorder Questionnaire (MDQ)

**DOI:** 10.3390/jcm13154433

**Published:** 2024-07-29

**Authors:** Uta Ouali, Amina Aissa, Salsabil Rejaibi, Nada Zoghlami, Amine Larnaout, Yosra Zgueb, Mejdi Zid, Hajer Aounallah Skhiri, Goce Kalcev, Massimo Tusconi, Cesar Ivan Aviles Gonzales, Antonio Preti, Diego Primavera

**Affiliations:** 1 Department Psychiatry A, Razi Hospital La Manouba, Manouba 2010, Tunisiaamina.aissa.83@gmail.com (A.A.);; 2 Faculty of Medicine of Tunis, University of Tunis El Manar, Tunis 1007, Tunisia; salsabil.rejaibi@fmt.utm.tn (S.R.);; 3 Research Laboratory LR18SP03, Razi Hospital La Manouba, Manouba 2010, Tunisia; 4 National Institute of Health, Tunis 1007, Tunisia; 5 Department Psychiatry D, Razi Hospital La Manouba, Manouba 2010, Tunisia; 6 Laboratoire de Recherche en Epidémiologie Nutritionnelle (SURVEN), Institut National de Nutrition, Tunis 1007, Tunisia; 7 Department of Medical Sciences and Public Health, University of Cagliari, Monserato Blocco I (CA), 09042 Cagliari, Italy; 8 University Hospital of Cagliari, 09042 Cagliari, Italy; massimotusconi@yahoo.com; 9 Department of Neuroscience, University of Turin, 10124 Turin, Italy

**Keywords:** hyperactivity, biological rhythm dysregulation, bipolar disorder, mood disorder, social rhythm dysregulation, stress, advances technologies laboratory

## Abstract

**Background:** This study aims to verify the accuracy of item 10 on the energy level of the Short-Form Health Survey (SF-12) in an item screening according to Mood Disorder Questionnaire (MDQ) results, providing a measure of hyper-energy. **Methods:** Regression techniques were employed in a dataset comprising 4093 records of respondents to test both linear and nonlinear relationships between predictor and outcome variables (energy level and symptoms considered in the MDQ). We examined the relationship of energy level with cases identified using MDQ with a cut-off of 7. **Results:** Levels of energy, as rated on item 10 of the SF-12, were related to the MDQ score with sensitivity = 0.72 and specificity = 0.70. In linear regression, the associations were stronger with MDQ items on excessive energy or activity, showing a medium effect size and an explained variance of 10% or higher. A greater association was observed for items on excessive energy and activity, as expected, as well as for items concerning self-confidence, sociability, and talkativeness. **Conclusions:** This result may have implications for the research on risk factors and the pathogenesis of the dysregulation of mood, energy, and social rhythms syndrome (DYMERS), a syndrome that is hypothesized to occur in stressful conditions like those shown under the COVID-19 pandemic.

## 1. Introduction

The accuracy of paper-and-pencil screeners for identifying individuals most likely to suffer from bipolar disorder has been the subject of heated debate [1,2]. In fact, it emerged that the use of these tools, particularly the Mood Disorder Questionnaire (MDQ) [3], was inaccurate, characterized by the identification of an excessive number of false positives. Moreover, people who presented false positives on the MDQ often had psychiatric disorders other than bipolar disorder, such as anxiety, addictions, personality disorders, and attention-deficit/hyperactivity disorder (ADHD) [4,5]. Some researchers, however, contrasted these conclusions by stating that the threshold commonly used by conventional diagnostic systems was too low to include the entire “bipolar spectrum” [6,7,8]. Furthermore, the disorders identified in people with “false positivity” were well-known to be frequently associated with bipolar disorder, and, often, the following disorders were found to be antecedent to the onset of bipolar disorder [9]: borderline personality disorders [10,11,12]; post-traumatic stress disorder [13,14]; phobic disorders [15,16]; alcohol disorders [17]; eating disorders [18,19]; impulse control disorder [20,21]; drug disorders [22]; and attention deficit disorder [23]. A study found that patients with major depressive disorders had abnormal resting brain activity in some brain areas compared to controls without major depressive disorders. People with major depressive disorders screened positive in a paper-and-pencil test for bipolar disorders, namely, the Hypomania Checklist (HCL-32) [24,25]; people without a clinical diagnosis of bipolar disorders had significantly different brain activity from people screening negative within, including the right orbitofrontal cortex, anterior cingulate cortex, gyrus rectus, and left inferior parietal cortex [26]. Another important finding from the large population studies was that a positive MDQ result was often associated with significant impairment and a notable decrease in perceived quality of life, even in the absence of any psychiatric diagnosis [27]. In light of these outcomes, we began to believe that the positivity of the MDQ could identify an area of non-specific suffering characterized by basic hyperactivity/hyper-energy with associated dysregulation of social rhythms and stress symptoms and the severe impairment of health-related quality of life even in the absence of a psychiatric diagnosis [28]. It would be a condition linked to prolonged stress, not related to any psychiatric diagnosis, but capable of evolving into different conditions (including bipolar disorder) based on individual vulnerability [29]. It has also been hypothesized that a specific personality characteristic/trait of hyperactivity/hyper-energy could be an antecedent of the dysregulation syndrome [30]. This trait may be related to some genetic variants previously thought to be associated with bipolar disorder but found equally in people with hyper-energy/hyperactivity and novelty-seekers without bipolar disorder [31]. Moreover, based on the evidence, the limitations during the COVID-19 pandemic lockdown, in which rhythms of life have been compromised, can influence the course of all those psychiatric vulnerabilities particularly susceptible to the dysregulation of biological rhythms. In other words, a rigid lockdown may cause a depressive relapse in these patients by disrupting their biological rhythms [32]. On the other hand, sleep disorders, biological rhythm disruptions, and work- or family-related stressors, which are more expressed in certain situations like the COVID pandemic, can serve as triggers for hypomanic episodes in bipolar disorder patients [33] or hyperactivity features in individuals without a mental illness [30].

From this perspective, it could be useful to analyze cohorts of people with, but above all without, any psychiatric disorders to understand whether some people can develop rhythm dysregulation syndrome. Another interesting element would be to know whether the hyperactivity/hyper-energy traits manifested prior to the syndrome are associated with the risk. In various fields of research, there are cohorts that have already been studied for a long time on individuals subjected to stressful conditions; for example, cohorts of patients with chronic non-psychiatric pathologies, workers in demanding professions, etc. When many of these studies were planned, it was far from thought that a trait of hyper-energy/hyperactivity could constitute a risk factor. While it is rare for a cohort on the effect of stress to have adopted an instrument for measuring hyper-energy/hyperactivity, it is common for this type of study to monitor the level of perception of quality of life. For this purpose, one of the most used instruments is the 12-Item Short Form Survey (SF-12) [34,35,36]. Item 10 of the SF-12 scale asks whether the respondent has felt full of energy in the last 4 weeks. The same question corresponds to item 23 of the 36-item Short Form Health Survey (SF-36) scale [37,38], another quality of life instrument more extensive than the SF-12, which is also widely used.

The aim of this work is to verify whether scores on item 10 of the SF-12 are a reliable marker of hyper-energy based on the results of the MDQ questionnaire. If this were verified, it would be possible to obtain a measure of hyper-energy that can be used in the analysis of historical cohorts to verify the genesis of the proposed dysregulation of mood, energy, and social rhythms syndrome (DYMERS) and its relationship with the risk of bipolar disorder.

## 2. Materials and Methods

### 2.1. Participants, Data Collection, and Ethical Aspects

**Figure 1:**
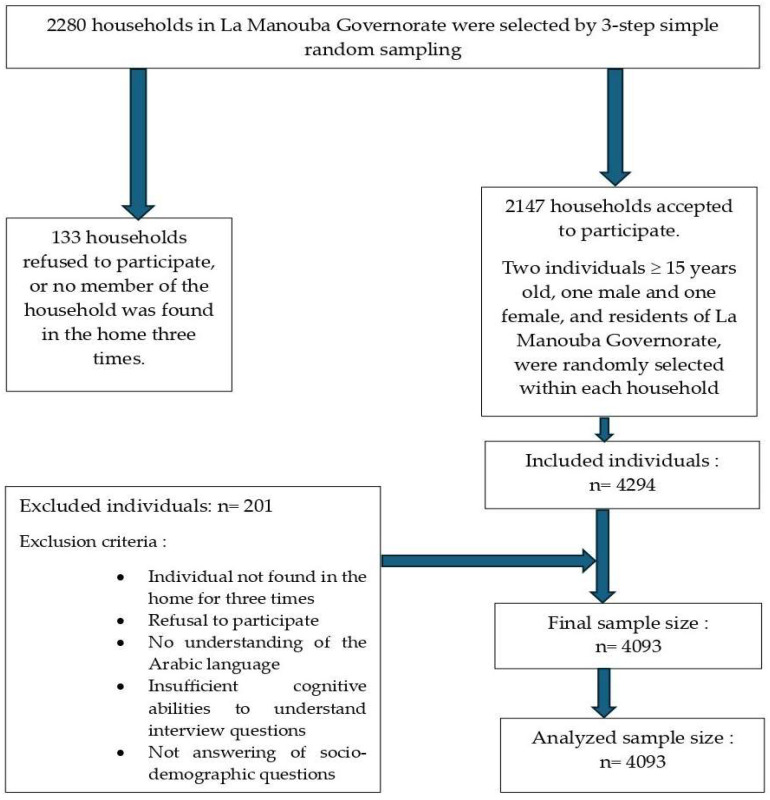
Flow chart of the participant selection process.

The survey was carried out among a random sample of households in the La Manouba governorate. Included were individuals ≥15 years old who were residents of the La Manouba governorate who understood the Arabic language and had sufficient cognitive abilities to understand the interview questions. Individuals living in military camps, prisons, or hospitals or residing only temporarily in a household were excluded, as were individuals who did not answer the socio-demographic questions of the survey. The sample size was calculated using a formula for simple random sampling described in detail in the protocol paper for the survey [39]. The required sample size was about 4540 individuals. The random sampling process was conducted by the Tunisian National Institute of Statistics in three steps: in the first step, 152 districts were selected among all districts of the governorate; in the second step, 15 households were selected per district; in the third step, two individuals aged 15 and over (one male and one female) were selected per household. The final number of individuals included was 4093 (Figure 1). 

The study protocol adhered to the 1995 Declaration of Helsinki and its subsequent revisions [40]. Participants provided signed informed consent, and the study received approval from (1) the Institutional Review Board of Razi University Hospital (RPA6/2021 from 17 June 2021); (2) the Research Directorate of the Tunisian Ministry of Health as well as the Regional Directorate of Health of La Manouba Governorate (from 11 November 2019); (3) the Tunisian National Instance of Protection of Personal Data (Number 20/02-4872 from 18 March 2020); and (4) the Tunisian National Council of Statistics (Number 21/04 from 24 September 2021).

### 2.2. Instruments

A semi-standardized interview was administered to consenting participants, enquiring about socio-demographic characteristics (including sex, age, marital status, and residence) and health conditions, including symptoms indicative of a mood disorder. The Arabic version of the Structured Clinical Interview for DSM IV-TR, modified for the World Mental Health Survey reappraisal interview, was used in this study [41]. Participants also completed a validated Arabic-Tunisian version of the MDQ and the similarly validated Arabic-Tunisian 12-item Short Form Health Survey (SF-12).

The Mood Disorder Questionnaire (MDQ) is a screening tool designed to detect symptoms of bipolar disorder [3]. It consists of a series of yes/no questions focusing on mood, behavior, and thought patterns commonly associated with bipolar disorder. The questionnaire aims to identify individuals who may benefit from further assessment for bipolar disorder based on their responses. The MDQ typically utilizes a specific scoring threshold to determine if someone is at risk for bipolar disorder. A commonly used scoring threshold to determine if someone is at risk for bipolar disorder is a positive response to seven or more out of the first thirteen items on the questionnaire [42]. This threshold suggests a higher likelihood of bipolar disorder and indicates the need for further evaluation by a mental health professional. Evidence of the reliability, validity, and discriminant capacity of the Arabic-Tunisian version of the MDQ has been provided [43,44].

The SF-12 is a shortened version of the 36-item Short Form Health Survey (SF-36) and includes a subset of questions from the SF-36 to assess health-related quality of life. It comprises 12 items covering physical and mental health domains, including aspects such as physical functioning, role limitations due to physical and emotional health, bodily pain, general health perceptions, vitality, social functioning, and mental health [36]. The SF-12 is a widely used self-reported questionnaire designed to assess health-related quality of life and exhibits adequate equivalence with the SF-36 across a broad range of disease populations, countries, and administration modes [45]. For this study, the SF-12 was adapted from the SF-36 scale validated in Standard Arabic [46].

### 2.3. Data Analysis 

Data were analyzed with the Statistical Package for Social Sciences (SPSS) version 27 and dedicated packages running in R [47]. All tests were two-tailed, with alpha set at *p* < 0.05. Means with a standard deviation or counts and percentages were used to describe the data. For descriptive purposes, Student’s or Welch’s *t*-test, depending on variance, and analysis of variance (ANOVA) were used to compare continuous variables, and chi-square, with Yates correction when necessary, was used to compare nominal variables. The effect size of the differences in continuous measures was estimated with Hedges’ g, with the following threshold according to Cohen [29]: around 0.2 = small effect; around 0.5 = medium effect; 0.8 or larger = large effect.

For the main aim of this study, the responses to the first 13 items of the MDQ and item number 10 of the SF-12 were used. The items of the MDQ are rated yes = 1 or no = 0, with yes indicating the occurrence of the symptom described by the item. Item 10 of the SF-12 inquiries about energy over the past four weeks with this question: “Did you have a lot of energy?” Responses are graduated on a 6-point Likert scale from “all of the time” (6) to “none of the time” (1).

Assuming a linear relationship between energy, as rated on item 10 of the SF-12, and the symptoms considered in the MDQ, a linear regression of item 10 of the SF-12 was applied to each item of the MDQ. In linear regression, the adjusted R^2^ can be considered a measure of the variance explained by the model. The standardized Beta of the linear regression can be interpreted similarly to a Pearson correlation coefficient and assessed in terms of effect size. The following thresholds were applied: around 0.10 indicates a small effect, around 0.30 indicates a medium effect, and around or above 0.50 indicates a large effect [48].

Given the binary (yes/no = 1/0) nature of the responses on the MDQ, we also tested the relationship between item 10 of the SF-12 and each item of the MDQ with a logistic regression. In logistic regression, the Nagelkerke pseudo-R² was calculated and interpreted analogously to the adjusted R² of the linear regression as a measure of the variance explained by the model. The odds ratio (OR) with a 95% confidence interval (CI) has been used as a measure of the effect size and interpreted as such: for each point increase in the predictor (item 10 of the SF-12), the OR expresses the increase in the chance of the occurrence of the symptom on the MDQ. For example, an OR = 2 implies that for each increase in point in the level of energy (from 1 to 6, as rated on item 10 of the SF-12), there is a doubling in the chance of the occurrence of the symptom on the MDQ.

We also tested the relationship between item 10 of the SF-12 and cases identified at the MDQ as at-risk of bipolar disorder based on the cut-off of 7. The receiver operating characteristics (ROC) curve analysis was applied to test the capacity of item 10 of the SF-12 to discriminate cases at risk of bipolar disorder, as identified using the MDQ from non-cases. The following parameters were reported: sensitivity (the probability of identifying an “at risk of bipolar disorder” case in the sample), specificity (the probability of identifying non-cases), positive predictive value (PPV or precision, the proportion of true cases out of all positive test results), negative predictive value (NPV, the proportion of true negative cases out of all negative results), and the area under the curve (AUC) with a 95%CI. The AUC was rated as poor (≤0.70), fair (0.70 to 0.80), good (0.80 to 0.90), or excellent (>0.90) according to Hosmer and Lemeshow [49]. The “pROC” package [50] and the “OptimalCutpoints” package [51] were used in R version 4.4 to perform the ROC analysis.

## 3. Results

**Figure 2:**
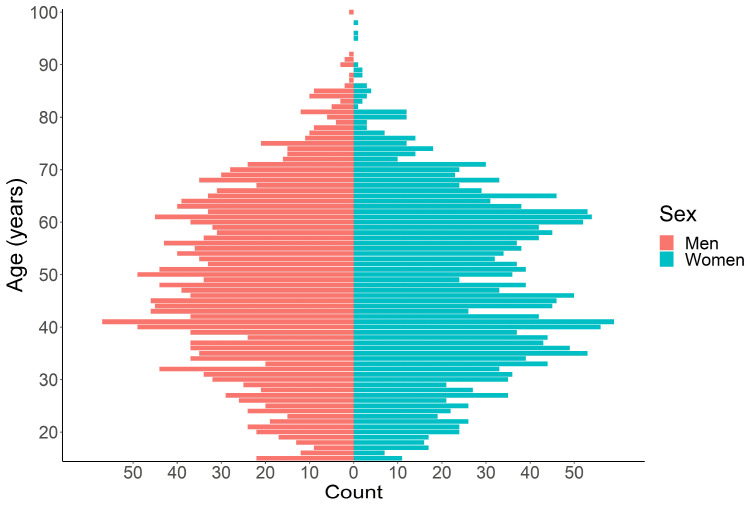
Pyramid of age.

Table 1 presents the main socio-demographic characteristics of the sample. The study included 4093 participants with a balanced distribution of sexes. The age of participants ranged from 15 to 100 years, with 235 participants (5.7%) aged 21 years or younger and 616 participants (15.1%) aged 66 years or older (Figure 2). The mean age of the sample was 47.7 ± 16.4 years. The majority of participants resided in urban areas.

The plot represents the age structure of the participating sample, indicating the number of persons (count) by block of age (in years) in the two sexes (men and women).

### 3.1. Responses to the Mood Disorder Questionaire (MDQ)

The reliability of the MDQ in the sample was excellent (Cronbach’s α = 0.90). A positive “yes” response ranged from 4% (item 11, more interested in sex than usual) to 14% (item 2, so irritable as to shout at people or start an argument). Scores on the first 13 diagnostic items of the MDQ ranged from 0 to 13, with a substantial portion of participants (*n* = 2906 [71%]) scoring 0 (no occurrence of symptoms in the past). Overall, 247 participants scored 7 or more on the MDQ, indicating they were “at risk of bipolar disorder”. Individuals scoring above the cut-off on the MDQ were more often men, single, and living in an urban setting compared to those who did not surpass the cut-off (Table 1).

### 3.2. Scores on Item 10 of the SF-12 (Levels of Energy)

**Figure 3:**
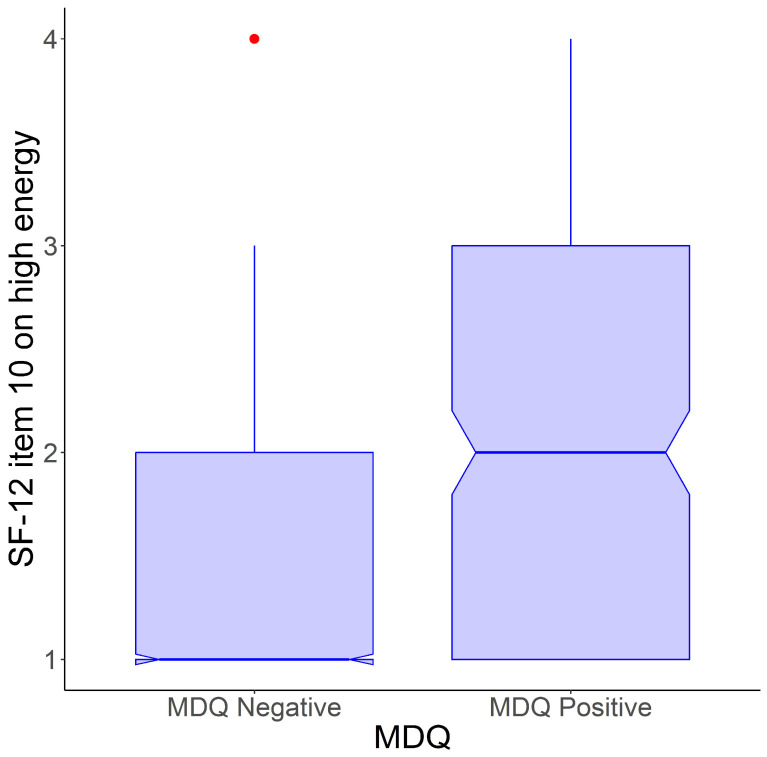
Distribution of scores of item 10 of the SF-12 (on high energy) by positivity on the MDQ according to the agreed cut-off.

In the sample, scores on item 10 of the SF-12 ranged from 1 to 4, with a mean of 1.5 (standard deviation = 0.7). There were no associations between levels of energy, as rated on item 10 of the SF-12, and sex or residency. Age was negatively related to scores on item 10 of the SF-12 with a small effect size (Pearson’s r = 0.10; *p* < 0.001). Separated or divorced individuals scored modestly higher on item 10 of the SF-12 compared to other marital status groups: singles = 1.5 ± 0.8; married = 1.4 ± 0.7; separated/divorced = 1.7 ± 1.0; widowed = 1.3 ± 0.6 (F[3, 4030] = 9.7; *p* < 0.001). Participants who scored above the cut-off for being “at risk of bipolar disorder” on the MDQ also scored significantly higher on item 10 of the SF-12 than those who did not surpass the cut-off: 2.1 ± 0.9 versus 1.4 ± 0.7; Welch’s *t*-test: 12.4 (d.f. = 266.0); *p* < 0.001; Hedges’ g = 1.0 (95% CI: 0.9–1.1) (Figure 3).

### 3.3. Relationship of Item 10 of the SF-12 with the Items on the MDQ

Levels of energy, as rated on item 10 of the SF-12, were statistically related to bipolar disorder symptoms as rated on the MDQ. In linear regression, the associations were stronger with MDQ items on excessive energy or activity, showing a medium effect size and an explained variance of 10% or higher (Table 2). The analysis with logistic regression showed an increase in explained variance and, in general, ORs of 2 or higher for most symptoms rated on the MDQ. A greater association was observed for items on excessive energy and activity, as expected, but also for items concerning self-confidence, sociability, and talkativeness (Table 3).

### 3.4. ROC Analysis of Item 10 of SF-12 on Risk of Bipolar Disorder as Detected Using MDQ

**Figure 4:**
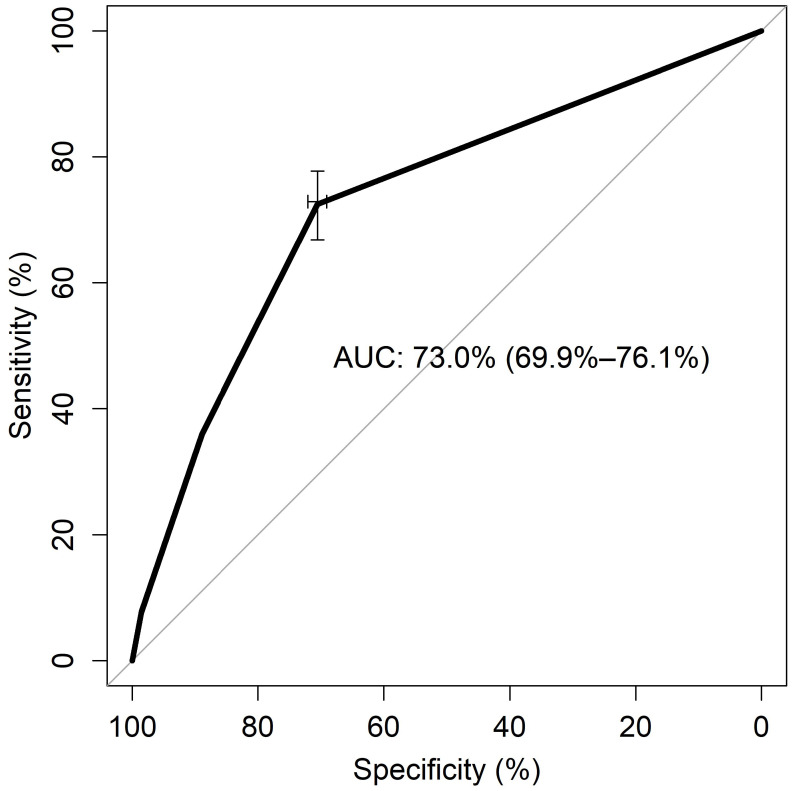
Receiver operator characteristic (ROC) curve of SF-12 item 10 against Mood Disorder Questionnaire (MDQ) positivity as Gold Standard. Sensitivity and specificity are reported as percentages, with a cross on the curve indicating the best compromise between them. The area under the ROC curve (AUC) is reported with its 95% confidence interval.

Levels of energy, as rated on item 10 of the SF-12, predicted the “positivity at MDQ” or, in another point of view, “hyperactivity sub-syndrome” (Figure 4). AUC was reasonably fair (73%), with sensitivity = 0.72 and specificity = 0.70. As expected for a study in the general population, item 10 of the SF-12 was more able to identify non-cases (NPV = 97%) than detect cases at risk of bipolar disorder (PPV = 13%). The identified best threshold for discrimination was 2, meaning that just a modest increase in the perceived levels of energy is associated with a greater chance of being positive for the risk of bipolar disorder or for “hyperactivity sub-syndrome” on the MDQ.

## 4. Discussion

The study shows that, in a large sample from the general population of the La Manouba governorate in Tunisia, the energy levels rated on item 10 of the SF-12 are statistically and reliably related to hyper-energy hyperactivity as rated on the MDQ. We tested both linear and nonlinear relationships between the predictor and outcome variables. In linear regression, the relationship between the predictor (here, scores on item 10 of the SF-12) and the dependent variable (each item of the MDQ) is expected to be straight. Accordingly, the slope of the fitted line may be equated to the correlation between the predictor and the dependent variable corrected by the ratio of the standard deviations of these variables. The advantage of linear regression is the opportunity to have an estimate of the explained variance of the relationship, based on the R^2^ and the adjusted R^2^, and to produce a simple explanation of their link. The beta coefficient is, indeed, the increase in the dependent variable for each point increase in the predictor. In the linear regression model, we found that there is a positive link between the levels of high energy, as measured by item 10 of the SF-12, and the occurrence of the symptoms rated on the MDQ. The effect size, based on the standardized beta, was small to moderate, with explained variance between 4% and 10% for most items of the MDQ. This is a reasonable indication of a link between the predictor and the dependent variables. However, the main limit of the linear regression model is the difficulty in deriving a linear increment in the dependent variable when the dependent variable is categorical, as in this case.

For this reason, we also resorted to a logistic regression model. A logistic regression model estimates the probability of an event occurring, in this case, the likelihood that a participant endorses a symptom on the MDQ as a function of the predictor (i.e., high energy levels measured by item 10 of the SF-12). Typically, the results are expressed as an odds ratio (OR), which is the odds that an outcome (symptom endorsing) will occur given a particular event (an increase in the levels of high energy), compared to the odds of the outcome occurring in the absence of that event. We found that the ORs were always statistically positive, meaning that an increase in high energy as rated on item 10 of the SF-12 was related to a high chance of symptom endorsing on each item of the MDQ, with explained variance between 5% and 16/17%, in general—better than in linear regression model.

The explained variance suggests an association between high levels of energy, as measured by item 10 of the SF-12, and symptoms rated on the MDQ, including mood dysregulation, energy dysregulation, and social disinhibition. High energy levels, as rated by item 10 of the SF-12, predicted positivity on the MDQ based on the agreed cut-off. Specifically, a lack of excessive energy predicted non-positivity on the MDQ, indicating that indicators of stress, hyperactivity, and rhythm dysregulation syndrome are related to subjective excessive energy rather than merely increased energy. This result may have implications for the research of risk factors and pathogenesis of DYMERS. In fact, it is a syndrome that is hypothesized to occur in stressful conditions and even in the absence of previously recognized psychiatric diagnoses. In fact, it is a syndrome that is hypothesized to occur in stressful conditions and even in the absence of previously recognized psychiatric diagnoses [52].

The COVID-19 pandemic has significantly impacted the lives of many individuals, leading to heightened concerns about contagion, bereavement, economic hardship, and subsequent increases in mood disorders across various communities [53,54,55,56]. Particularly vulnerable or high-risk populations have experienced exacerbated effects [57,58]. The lockdown measures, but especially the stressful condition of the pandemic, have had a significant effect in contributing to the dysregulation of personal and social rhythms closely linked to biorhythms, posing additional risks, notably for individuals particularly susceptible to the mood axis, although not necessarily having a diagnosis.

It is, therefore, important to study large cohorts over time not characterized by psychiatric disorders, with attention to people subjected to stressful conditions such as workers in demanding or risky professions [59,60] not only during the pandemic but also in the immediate subsequent phases [61], patients with chronic illness [62,63,64], people subjected to wars or forced migrations [65,66], people subjected to environmental stress such as those capable of modifying bio-rhythms such as strong light and noise pollution [67,68,69]. In this way, it will be possible to verify, as hypothesized on the basis of previous studies on small samples, the pathological path that predicts that hyperactive people who are not necessarily carriers of one pathology/disorder under intense and prolonged stress, for example, like those of the COVID-19 pandemic, can develop the syndrome of dysregulation of rhythms and hyperactivity and that this condition may be the step towards bipolar disorder and/or other pathologies based on individual pre-disposition. A screening test consisting of just one item is unlikely to be useful as a case-finder due to its difficulty and tendency to identify many false positives [70,71]. However, in large cohorts over time, it can be useful for studying risk conditions that, although diluted by false positives, should be considered. Additionally, it should be noted that an extremely streamlined screening tool, despite its potential risks, allows for easy monitoring of health conditions in large population samples.

One of the limitations of this study could be the inclusion of households in only one (La Manouba governorate) out of 24 provinces in Tunisia. This limitation in sample selection may constrain the generalizability of the results, not in relation to the psychometric properties of the measurement instruments but rather concerning the implications for understanding in greater detail the risk factors and underlying mechanisms affecting the alteration of mood, energy, and social rhythms.

The further implications for implementing future public health policy are that interest in this new syndrome (DYMERS) derives from its seeming crucial role in the exacerbation of chronic conditions, representing a distinct clinical profile associated with stress. This underscores the importance of social rhythms in stress prevention and their potential implications for health and well-being. DYMERS could be configured as a condition of vulnerability common to various syndromes, including panic disorder (PD), attention deficit hyperactivity disorder, post-traumatic stress disorder, and others.

## 5. Conclusions

This study highlights the potential of using item 10 of the SF-12 as a reliable measure for hyper-energy that is well associated with MDQ results including mood dysregulation, energy dysregulation, and social disinhibition, particularly in vulnerable populations. Despite the limitations of single-item screening tools, their simplicity can facilitate the monitoring of large cohorts over time. This approach may be valuable in understanding the genesis of dysregulation of mood, energy, and social rhythms syndrome (DYMERS), offering insights into stress-related conditions even in the absence of prior psychiatric diagnoses.

## Figures and Tables

**Table 1:** Socio-demographic characteristics of the sample (*n* = 4093).

	Global Sample	Negative on the MDQ	At-Risk on the MDQ	Statistics
	*n* = 4093	*n* = 3840	*n* = 247	
Sex				χ^2^ = 12.4; δϕ = 1; π < 0.001
Men	2001 (48.9%)	1849 (48.2%)	148 (59.9%)	
Women	2092 (51.1%)	1991 (51.8%)	99 (40.1%)	
Age	47.7 (16.4)	48.6 (16.5)	47.9 (15.2)	Welch’s t = 0.8; df = 417.5; *p* = 0.40
Marital status				χ^2^ = 37.5; δϕ = 3; π < 0.001
Single	751 (18.3%)	671 (17.7%)	79 (32.5%)	
Married	2981 (72.8%)	2827 (74.6%)	151 (62.1%)	
Separated/Divorced	62 (1.5%)	56 (1.5%)	6 (2.5%)	
Widow/Widower	245 (6.0%)	238 (6.3%)	7 (2.9%)	
Missing/Undeclared	54 (1.3%)			
Residency				χ^2^ = 8.2; δϕ = 1; π = 0.004
Urban	3189 (77.9%)	2972 (77.4%)	211 (85.4%)	
Rural	904 (22.1%)	868 (22.6%)	36 (14.6%)	

All data: *n* (%) or mean (standard deviation).

**Table 2:** Results of linear regression of item 10 of SF-12 on each of the first 13 items of the MDQ.

Item	R^2^	adj. R^2^	Beta (s.e.)	St. Beta	t	*p*
1. So hyper as to get into trouble	3.2%	3.2%	0.05 (0.01)	0.18	11.6	<0.001
2. So irritable as to start an argument	0.1%	0.1%	0.04 (0.01)	0.10	6.2	<0.001
3. More self-confident than usual	7.6%	7.6%	0.11 (0.01)	0.28	18.4	<0.001
4. Less need for sleep	4.3%	4.3%	0.08 (0.01)	0.21	13.5	<0.001
5. More talkative than usual	4.4%	4.4%	0.08 (0.01)	0.21	13.8	<0.001
6. Racing thoughts	4.6%	4.5%	0.09 (0.01)	0.21	13.9	<0.001
7. Difficulties in concentrating	2.8%	2.8%	0.08 (0.01)	0.17	10.8	<0.001
8. More energy than usual	10.5%	10.5%	0.14 (0.01)	0.32	21.9	<0.001
9. More active than usual	11.0%	11.0%	0.13 (0.01)	0.33	22.4	<0.001
10. More social or outgoing than usual	4.9%	4.8%	0.08 (0.01)	0.22	14.4	<0.001
11. More interested in sex than usual	2.1%	2.0%	0.04 (0.01)	0.14	9.3	<0.001
12. Too foolish or risky	2.0%	2.0%	0.04 (0.01)	0.14	9.1	<0.001
13. Spending more money than usual	2.5%	2.5%	0.04 (0.01)	0.16	10.3	<0.001

adj. = adjusted; s.e. = standard error; St. = Standard.

**Table 3:** Results of logistic regression of item 10 of SF-12 on each of the first 13 items of the MDQ.

Item	R^2^ Nagelkerke	OR (95%CI)	Wald Test	Wald *p*
1. So hyper as to get into trouble	7.2%	2.1 (1.8–2.4)	115.7	<0.001
2. So irritable as to start an argument	1.5%	1.4 (1.2–1.5)	36.8	<0.001
3. More self-confident than usual	12.4%	2.5 (2.4–2.8)	260.2	<0.001
4. Less need for sleep	7.7%	2.1 (1.9–2.4)	155.6	<0.001
5. More talkative than usual	8.2%	2.2 (1.9–2.4)	159.0	<0.001
6. Racing thoughts	7.0%	1.9 (1.8–2.2)	169.0	<0.001
7. Difficulties in concentrating	4.4%	1.7 (1.5–1.9)	107.2	<0.001
8. More energy than usual	16.0%	2.8 (2.5–3.1)	342.8	<0.001
9. More active than usual	17.0%	2.9 (2.6–3.3)	351.5	<0.001
10. More social or outgoing than usual	9.3%	2.3 (2.0–2.6)	170.3	<0.001
11. More interested in sex than usual	5.8%	2.0 (1.7–2.4)	75.2	<0.001
12. Too foolish or risky	5.4%	2.0 (1.7–2.3)	73.2	<0.001
13. Spending more money than usual	6.2%	2.1 (1.8–2.4)	91.3	<0.001

OR = odds ratio; CI = confidence interval.

## Data Availability

The datasets of this study will not be publicly available due to individual privacy rules.
